# Exploring pain management in breast cancer: key findings from the ARISE study

**DOI:** 10.3389/fonc.2025.1536709

**Published:** 2025-03-12

**Authors:** Costanza M. Donati, Alice Zamagni, Arina A. Zamfir, Cynthia Aristei, Silvia Cammelli, Claudio Zamagni, Silvia Paolinelli, Milly Buwenge, Romina Rossi, Marco Maltoni, Alessio G. Morganti, Savino Cilla

**Affiliations:** ^1^ Radiation Oncology, Istituti di Ricovero e Cura a Carattere Scientifico (IRCCS) Azienda Ospedaliero-Universitaria di Bologna, Bologna, Italy; ^2^ Department of Medical and Surgical Sciences (DIMEC), Alma Mater Studiorum University of Bologna, Bologna, Italy; ^3^ Radiation Oncology Unit, Clinical Cancer Centre, AUSL-Istituti di Ricovero e Cura a Carattere Scientifico (IRCCS) di Reggio Emilia, Reggio Emilia, Italy; ^4^ Radiation Oncology Section, University of Perugia and Perugia General Hospital, Perugia, Italy; ^5^ Addarii Medical Oncology, Istituti di Ricovero e Cura a Carattere Scientifico (IRCCS) Azienda Ospedaliero-Universitaria di Bologna, Bologna, Italy; ^6^ Palliative Care Unit, Istituti di Ricovero e Cura a Carattere Scientifico (IRCCS) Istituto Romagnolo per lo Studio dei Tumori (IRST) “Dino Amadori”, Meldola, Italy; ^7^ Medical Oncology Unit, Department of Medical and Surgical Sciences (DIMEC), University of Bologna, Bologna, Italy; ^8^ Medical Physics Unit, Responsible Research Hospital, Campobasso, Italy

**Keywords:** breast cancer, pain management, radiotherapy, patient outcomes, predictive variables, ARISE study, healthcare disparities, pain measurement

## Abstract

**Aims:**

This ARISE study secondary analysis aims to delve into the complexities of pain management in breast cancer patients undergoing radiotherapy (RT) in Italy. It aims to identify and analyze predictive variables for pain management adequacy and establish the relationship between these variables and the effectiveness of pain control.

**Materials and methods:**

This observational study engaged 2,104 participants from 13 Italian RT departments, focusing on 426 breast cancer patients reporting pain. Advanced statistical methods, were employed to identify significant predictive variables for pain management adequacy. Data collection involved a standardized form capturing personal, health-related information, specifics about cancer, pain intensity, and medication. The Pain Management Index (PMI) was used to evaluate pain management adequacy, where negative PMI values indicate inadequate or suboptimal pain management.

**Results:**

The analysis showed that 61.7% of patients experienced inadequate pain management (PMI<0). Factors identified as influencing pain management adequacy included the type of pain, patient age, the objective of RT, and the geographical location of the RT center. Notably, patients undergoing curative RT exhibited a higher incidence of inadequate pain management (PMI<0) compared to those undergoing palliative RT (82.9% versus 31.4%). Geographical variations were evident, with patients treated in northern Italy showing better pain management compared to those in central-southern Italy (72.0% versus 85.6%).

**Conclusion:**

The ARISE study underscores a significant inadequacy in pain management among breast cancer patients undergoing RT in Italy, influenced by a complex interplay of treatment-related, demographic, and regional factors. The study findings emphasize the need for enhanced, personalized pain management strategies and highlight the importance of considering a multifaceted approach.

## Introduction

Pain significantly reduces the quality of life (QoL) in cancer patients, impacting their physical, psychological, and spiritual well-being (1–3). Furthermore, a negative impact on the QoL in cancer patients can also be caused by pain of non-neoplastic origin (4). Therefore, also proper treatment of this pain is relevant as managing cancer-related pain. However, it has been repeatedly observed that non-neoplastic pain therapy in cancer patients is frequently inadequate (4–6). Challenges to effective pain control encompass both the healthcare system and patient perspectives (7). These include a lack of knowledge and skills among healthcare professionals (8), and a hesitancy among patients to communicate their pain (9). Recognizing the critical role of pain management is vital for enhancing patient outcomes (10).

The widespread issue of pain has led to efforts to bolster educational programs in universities and ongoing professional training, focusing on supportive treatments (11, 12). Moreover, educating patients about pain has become increasingly recognized as a key component of effective pain management (13, 14). Nevertheless, pain associated with breast cancer (BCa) often receives insufficient attention, resulting in subpar management outcomes compared to other cancers (5, 15, 16). Our prior ARISE studies corroborate this pattern, showing a marked link between BCa and suboptimal pain management (6).

To address the unclear reasons behind the generally less effective pain management in BCa patients, we undertook a detailed sub analysis of the ARISE study (6), focusing on BCa patients dealing with pain in Italian radiotherapy (RT) centers. Utilizing advanced statistical techniques, we sought to unravel the connection between the adequacy of pain management and various factors, such as the geographic site of the RT center, the demographics of patients, and the characteristics of pain. Moreover, our study seeks to fill this gap by systematically analyzing pain management practices and outcomes, utilizing patient-reported data to ensure a comprehensive assessment. Unlike previous research, which often focuses on broad oncological populations or singular interventions, our work integrates a multifaceted approach to the quality of pain management.

Finally, Italy is conventionally divided into three macro-regions: North, Center, and South, which differ for cultural, economic, and social reasons. Our previous analysis on the ARISE study (6) had shown that the geographic location of the RT center had a very significant impact on the adequacy of pain therapy. Therefore, in the present analysis we wanted to verify whether this phenomenon was also recorded in the subgroup of patients affected by BCa.

## Materials and methods

### Study design

Our investigation was set up as a cross-sectional, observational study aimed at evaluating the prevalence of pain and the adequacy of its management in RT departments (6, 17). This analysis focused specifically on BCa patients reporting pain or those taking analgesic drugs regardless of reported pain.

### Setting

The study was conducted across 13 Italian RT departments (**Table 1**). Patients were evaluated during their RT visits in October and November 2019.

**Table 1:** Participating centers in the ARISE study, categorized by geographic area, along with the number of patients enrolled in the analysis.

	Location of the radiotherapy center	Radiotherapy center	Number of patients
1	North of Italy	Bologna	50
2		Verona	10
3		Forlì	10
4	Center of Italy	Campobasso	15
5		Roma	29
6	South of Italy	Rionero in Vulture	30
7		Cosenza	34
8		Chieti	43
9		Messina	38
10		San Giovanni Rotondo	61
11		Bari	34
12		Brindisi	30
13		Napoli	42

### Participants

Inclusion criteria were as follows: (1) BCa cancer patients (regardless of tumor stage and RT aim), (2) treated in RT departments, and (3) aged ≥ 18 years. Patients with comorbidities (psychiatric disorders or neurosensory deficits) preventing data collection or informed consent were excluded. Eligible participants were those using analgesic drugs solely for pain management, excluding those on medications for non-pain-related purposes (e.g., opioids for cough sedation or dyspnea) (18).

### Variables

The primary outcome of the study was the adequacy of pain management, assessed using the previously validated Pain Management Index (PMI) (4, 5, 15, 16, 20–27). Predictive variables analyzed included patient demographics (age, ECOG-PS), cancer stage (metastatic vs. non-metastatic), nature of pain (cancer-related, non-neoplastic, or mixed), aim of RT, geographic location of the RT facility (North, Central, or South Italy), and timing of the visit (during RT or at the end of RT).

### Data sources/measurement

All patients who met the enrollment criteria and who underwent a clinical visit at least once in the RT departments of participating centers in the period October–November 2019 were included. The evaluation was performed regardless of the visit timing (ongoing RT visits or clinical evaluation at the end of treatment). However, each patient was evaluated only once. Data collection utilized a standardized form (**Supplementary Material 1**) capturing personal and health-related information, cancer specifics, pain intensity (measured using the Numeric Rating Scale, NRS), and medication details. The Pain Management Index (PMI) was used to evaluate pain management adequacy, where negative PMI values indicate inadequate pain management. Pain was categorized as cancer-related, non-cancer-related, or mixed based on clinical evaluation and diagnostic imaging. The intensity of pain was recorded as the average experienced by patients during the week before their assessment.

### Bias

Selection bias may have been introduced as participation was voluntary, potentially underrepresenting patients with more severe pain or those less engaged in care. Recall bias is also a possibility due to reliance on patient-reported data for pain intensity.

### Study size

**Figure 1:**
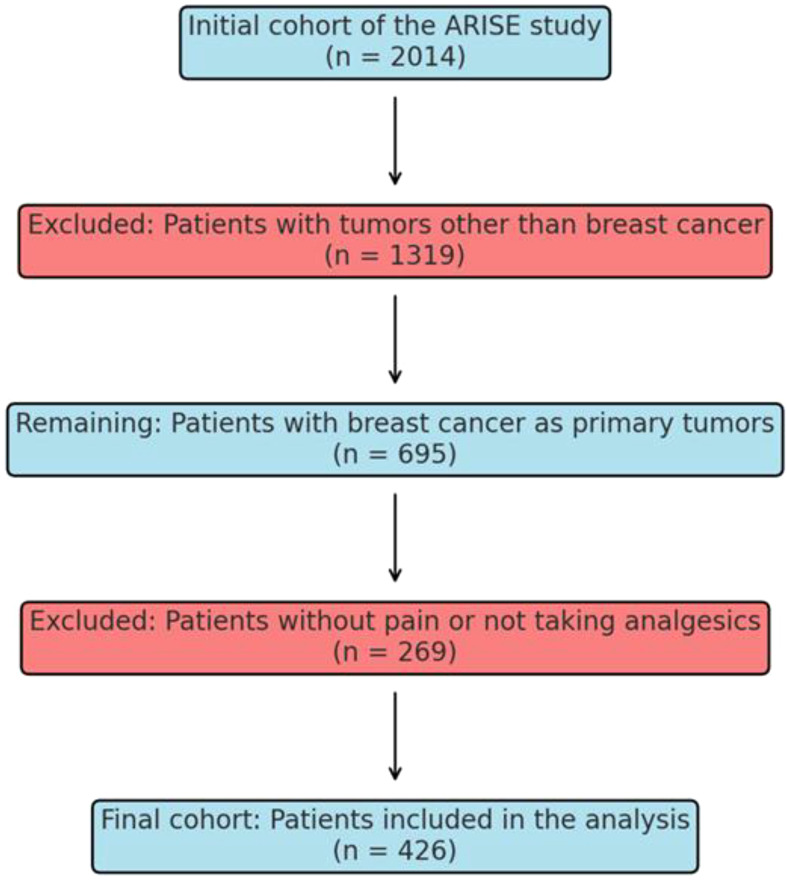
Flowchart of study cohort selection in the ARISE study.

The study engaged 2,104 participants across all RT departments, focusing on 426 BCa patients reporting pain (**Figure 1**). The sample size was deemed sufficient for statistical analysis and predictive modeling but may limit the ability to detect smaller clinically relevant effects.

### Quantitative variables

We assigned a pain score by using the following values: 0 (NRS: 0, no pain), 1 (NRS: 1–4, mild pain), 2 (NRS: 5–6, moderate pain), and 3 (NRS: 7–0, intense pain). In addition, based on the therapy the patients took, we defined an analgesic score as follows: no analgesics: 0, non-opioid analgesics: 1, “weak”opioids: 2, and “strong” opioids: 3. The Pain Management Index (PMI) was calculated by subtracting the pain score from the analgesic score, considering prescriptions with a negative value as inadequate (6, 17–19).

### Statistical methods

The Least Absolute Shrinkage and Selection Operator (LASSO) algorithm and Classification and Regression Tree (CART) analysis were employed to identify predictive variables and construct predictive models. LASSO filtered variables for inclusion in the model, and CART was used for decision tree-based modeling. Model robustness was ensured through cross-validation (5-fold, repeated 100 times), and predictive performance was evaluated using Receiver Operating Characteristic (ROC) curves and the Area Under the Curve (AUC) statistic.

## Results

**Figure 2:**
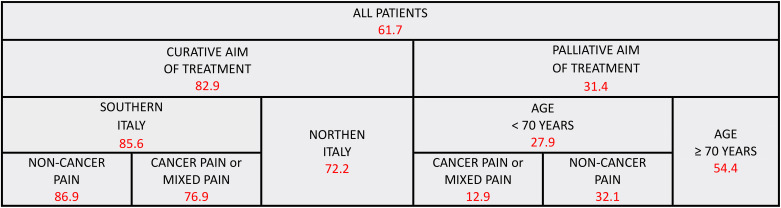
Predictive model for inadequate pain management: red numbers represent the proportion of patients with inadequate pain management (PMI < 0); all patients with breast cancer were included.

The ARISE study encompassed 2,104 participants across 13 Italian RT departments, with 1,387 individuals reporting pain or on analgesic medication. Among the latter, this analysis centered on 426 BCa patients, with demographic and clinical specifics outlined in **Table 2**. The PMI served as the tool to evaluate pain management effectiveness, indicating that 61.7% of the patients experienced suboptimal management. Employing LASSO analysis, we identified crucial determinants for a PMI below zero. Particularly, we identified the following variables as related to the study endpoint (PMI): type of pain, patient age, RT aim, and geographical location of the RT center (**Figure 2**). On the contrary, ECOG-PS, tumor stage, and timing of the assessment were not correlated with negative PMI values. In fact, the PMI assessment of pain management adequacy revealed significant disparities among patients and varied depending on the characteristics of the treatment.

**Table 2:** Patients characteristics (426).

		Number	(%)
Age, years			
≤ 70		315	74.0
71-80		90	21.0
> 80		21	5.0
ECOG-PS			
0-1		307	72.0
2		82	19.0
3		34	8.0
4		3	1.0
Aim of treatment			
Curative		251	59.0
Palliative		175	41.0
Tumor stage			
Metastatic		190	45.0
Non-Metastatic		236	55.0
Type of Pain			
Cancer Pain or mixed pain		216	51.0
Non-cancer Pain		210	49.0
Pain score			
(NRS: 0)	0	4	1.0
(NRS: 1 – 4)	1	198	46.5
(NRS: 5 – 6)	2	155	36.5
(NRS: 7 – 10)	3	69	16.0
Analgesic score			
(No therapy)	0	156	37.0
(Analgesics)	1	180	42.0
(Weak Opioids)	2	44	10.0
(Strong Opioids)	3	46	11.0
Location of the radiotherapy center			
Nord of Italy		70	16.0
Center of Italy		44	10.0
South of Italy		312	74.0
Timing of visit			
During Therapy		209	49.0
End of Therapy		217	51.0

Specifically, patients receiving curative RT exhibited a higher incidence of PMI<0 compared to those undergoing palliative RT (82.9% versus 31.4%). Additionally, within the curative RT group, a lower incidence of PMI<0 was observed in patients treated in northern Italian centers than in those from central-southern Italy (72.0% versus 85.6%). Within this latter group, patients experiencing non-neoplastic pain had a higher frequency of PMI<0 compared to those with neoplastic pain (86.9% versus 76.9%). Conversely, in the palliative RT cohort, a greater incidence of PMI<0 was noted in patients older than 70 years compared to their younger counterparts (54.4% versus 27.9%). Within this younger subgroup, those with mixed pain (both neoplastic and non-neoplastic) exhibited a lower rate of PMI<0 in contrast to those with solely neoplastic or non-neoplastic pain (12.9% versus 32.1%).

**Figure 3:**
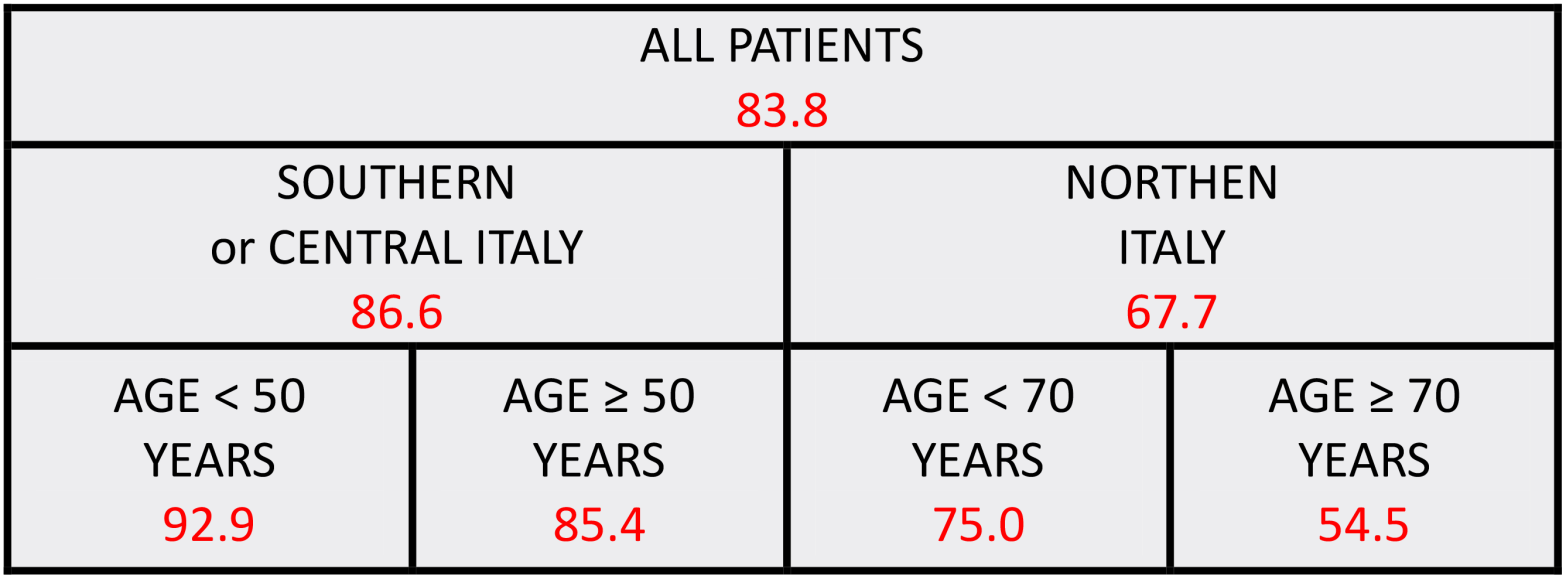
Predictive model for inadequate pain management: red numbers represent the proportion of patients with inadequate pain management (PMI < 0); only patients with breast cancer and non-neoplastic pain were included.

Furthermore, acknowledging the evidence of inferior pain management in cancer patients with non-neoplastic pain (4–6), a specialized analysis was conducted focusing solely on BCa patients with non-neoplastic pain. This analysis resulted in a model highlighting the geographical location of the RT center and age as significant factors influencing pain management adequacy (**Figure 3**). Specifically, patients treated in northern Italy demonstrated a lower incidence of PMI<0 in comparison to those in central-southern Italy (67.7% versus 86.6%). Within the latter group, patients younger than 50 years experienced a higher rate of PMI<0 relative to their older counterparts (92.9% versus 85.4%). However, among patients treated in northern Italy, those older than 70 years exhibited a lower incidence of PMI<0 compared to younger patients (54.5% versus 75.0%).

**Figure 4:**
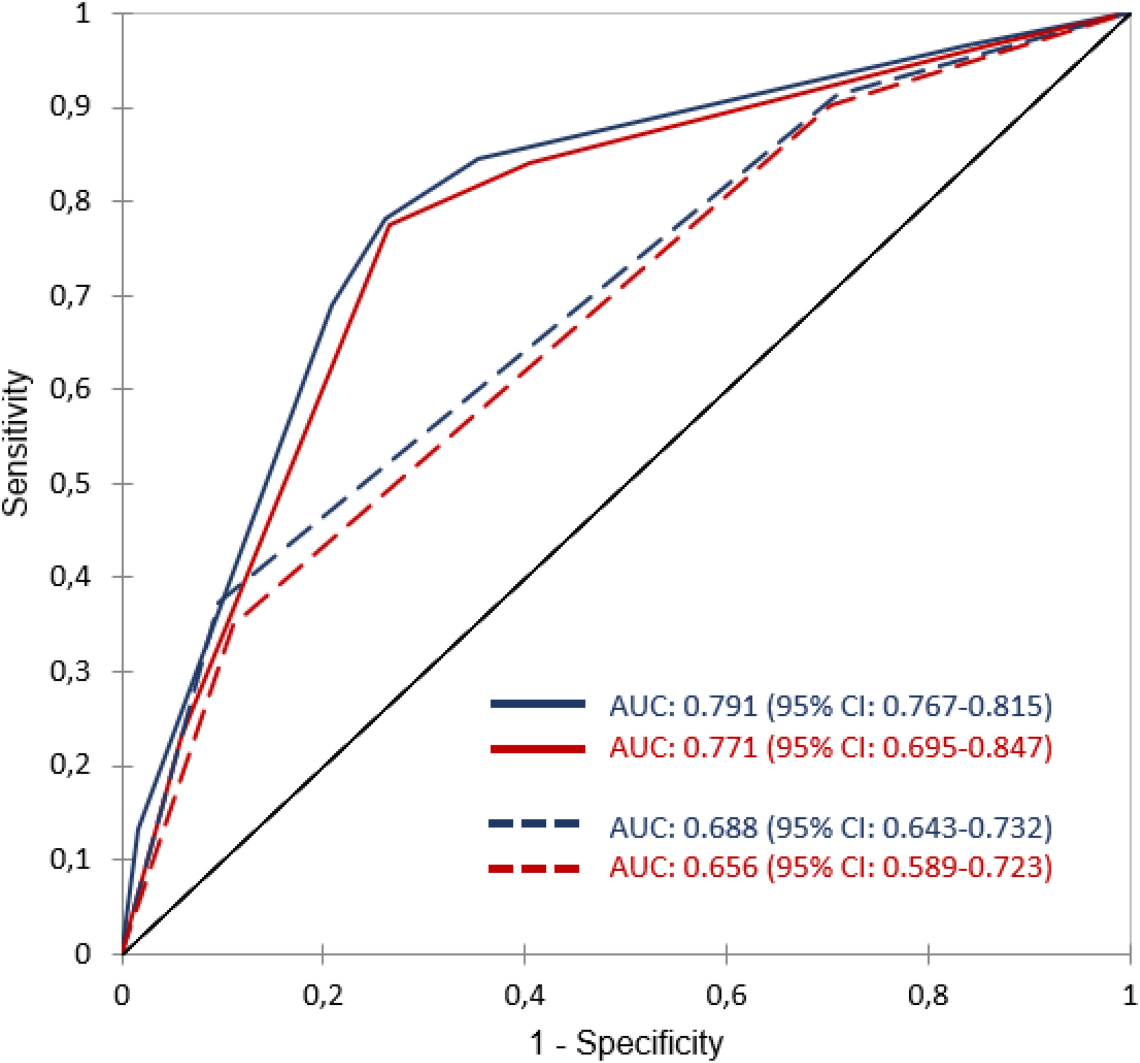
ROC curves and AUC values of the two CART models. The solid blue and red curves are the average ROC for the training and validation sets in the model including all patients. Dotted blue and red curves are the average ROC for the training and validation sets in the model including only patients with non-neoplastic pain.

The classification performances of the CART models are reported in **Figure 4** in terms of AUCs with 95% confidence intervals, demonstrating excellent generalizability. Considering all patients, the CART model showed AUC values of 79.1% (95%CI: 0.767-0.815) and 77.1% (95% CI: 0.695-0.847) in the training and validation datasets, respectively. With respect to BCa patients with non-neoplastic pain, the CART model showed AUC values of 68.8% (95% CI: 0.643-0.732) and 65.6% AUC: 0.656 (95% CI: 0.589-0.723) in the training and validation datasets, respectively.

## Discussion

The ARISE study comprehensive examination of pain management in BCa patients undergoing RT in Italy reveals a nuanced landscape of pain control efficacy. Despite the recognition of pain multifaceted impact on cancer patients’ lives, our findings underscore a prevalent inadequacy in pain management, with 61.7% of the studied cohort experiencing suboptimal care. The application of advanced statistical methods, including LASSO and CART analysis, has brought to light significant disparities in pain management adequacy.

The LASSO algorithm was chosen for its ability to perform both variable selection and regularization, which is critical in avoiding overfitting in datasets with high-dimensional predictors. By shrinking some coefficients to zero, LASSO identifies the most relevant variables influencing pain management adequacy, enhancing model interpretability. CART analysis complements LASSO by providing an intuitive and visual decision-making framework, allowing for the exploration of interactions between variables and the identification of thresholds critical for clinical decisions.

However, we acknowledge certain limitations of these methods. LASSO assumes linear relationships and may struggle to capture complex, non-linear interactions without appropriate transformation or feature engineering. CART, while interpretable, is prone to overfitting, particularly in the presence of noisy data. To mitigate these issues, we utilized cross-validation and pruning techniques to ensure model robustness and generalizability. These methods were selected to balance interpretability and predictive accuracy, aligning with the study’s goals to provide actionable insights for clinical practice.

The disparities recorded in our analysis are not random but are closely associated with age, nature of pain, RT aim, and geographical location of RT centers.

The lesser adequacy in the management of non-neoplastic pain may derive from concerns that treatment of chronic-benign pain with opioids could result in drug addiction, as well as from potentially less attention by physicians to symptoms not directly caused by cancer. We acknowledge the complexity in quantifying how these two factors specifically impacted our results. However, it is noteworthy to mention the stark contrast in inadequacy of non-neoplastic pain management between patients undergoing curative and palliative RT (86.9% vs 32.1%, respectively). Nevertheless, also this difference may stem both from greater physician attention to patients with advanced disease, irrespective of the pain origin, and from differing risks of opioid drug addiction in these two distinct patient populations.

Particularly striking is the difference in pain management effectiveness between patients undergoing curative and palliative RT, and the further influence of regional practices, as evidenced by the variations between northern and central-southern Italian centers.

Our analysis aligns with existing literature in several aspects, confirming the suboptimal pain management in BCa patients (5, 16, 20), particularly in those undergoing curative therapy compared to palliative therapy (6, 25), the inferior management of non-neoplastic pain (4–6), geographic disparities in pain management effectiveness (5, 6), and the adverse influence of younger age on pain management adequacy (6, 16). However, our findings indicate a complex relationship between age and PMI<0, differing from previous reports. While earlier studies suggested poorer pain management among younger patients (6, 16), our analysis across all pain types shows a higher incidence of PMI<0 among older patients (>70 years) receiving palliative RT. This could imply that healthcare professionals might prioritize pain management more in younger, symptomatic BCa patients, or it may reflect younger patients’ greater likelihood to report pain symptoms compared to their older counterparts. Additionally, it is important to consider that clinicians might often exhibit reluctance in prescribing opioids to older patients due to potential adverse effects, particularly cognitive impairment and increased risk of falls. These concerns can heavily influence prescribing practices, especially in contexts where the risk of these side effects might outweigh the benefits of pain relief.

Moreover, the complexity of pain management in patients under 70 years undergoing palliative RT is highlighted by the finding that pain management is more adequate in patients with mixed pain compared to those with only neoplastic or non-neoplastic pain. This contrasts with previous studies, which generally found an intermediate quality of pain management in patients with mixed pain (6).

Regarding the differential adequacy of pain management in patients undergoing palliative versus curative RT, we propose the following hypotheses: Firstly, it is plausible that in palliative care settings, clinicians prioritize quality of life, thereby focusing more attentively on symptom relief, including the provision of adequate pharmacological therapy. In contrast, the focus in curative treatment settings might lean more towards clinical outcomes, potentially at the expense of optimal symptom management. Secondly, patients receiving palliative RT might more frequently be under the care of clinicians specialized in supportive and palliative therapies, who are perhaps better experienced in prescribing effective pain management regimens.

The intricacies of pain management in BCa are further underscored by our secondary analysis focusing exclusively on patients with non-neoplastic pain. In fact, this study confirms better symptomatic treatment in patients in northern Italy, possibly due to superior clinical management by northern healthcare professionals or a reluctance among southern patients to report pain. This reluctance could be related to different psychological profiles shaped by varying socio-economic conditions (28–30).

In younger patient groups from both northern and central-southern Italy, poorer pain management was noted. The age threshold for this disparity was 70 years in the north and 50 years in the south, suggesting regional differences in pain management effectiveness between younger or middle-aged patients and older adults. This raises questions about whether these variations are due to different sensitivities of healthcare professionals towards patient age or if they reflect regional influences on patients’ psychological profiles, affecting their likelihood to report pain. Undoubtedly, additional research is needed to enhance the current unsatisfactory state of pain management in BCa patients. Specifically, it is crucial to discern the extent to which the documented disparities stem from the attitudes of healthcare providers versus those of the patients themselves. Pursuing this line of inquiry, future studies could incorporate the utilization of well-designed questionnaires. These instruments should be capable of differentiating between pain that patients spontaneously report during routine clinical interactions with their oncology healthcare providers, and pain revealed in response to specific, direct inquiries about the intensity and nature of pain posed by interviewers. This approach would provide a more nuanced understanding of the dynamics influencing pain reporting and management in the clinical setting.

The ARISE-breast study, with its extensive cohort of 426 participants across 13 Italian RT departments, offers a robust, observational insight into the complexities of pain management in BCa patients. A notable strength of our study is the application of advanced statistical methods, such as LASSO and CART analysis, which enabled the identification of significant predictive variables for pain management adequacy. This methodological rigor offers a comprehensive understanding of the multifactorial nature of pain management, encompassing patient demographics, pain characteristics, treatment objectives, and even geographical discrepancies in treatment practices.

However, the study is not without limitations. The observational nature of the study, while offering real-world insights, limits our ability to infer causality. The reliance on patient-reported outcomes for pain intensity and management adequacy may introduce subjective biases, potentially influenced by individual pain thresholds and communication barriers. Furthermore, the study utilized a single definition of pain (average pain). Additionally, the opioids prescribed were not categorized as long-acting or short-acting. The specific pharmacological agents used in the analgesic drugs were not documented, and there is no available information regarding which clinician or specialist prescribed the analgesic therapy. Moreover, the study focuses on a single country, Italy, which, while providing in-depth regional insights, may limit the generalizability of our findings to other healthcare systems with different cultural, socio-economic, and medical practice landscapes. In fact, to the best of our knowledge, this is the first study dedicated to the analysis of pain adequacy in BCa patients. This has limited our ability to compare with studies conducted in other settings (other than RT) or in other countries. Additionally, our study assessment was based on a single time point, evaluating a single pain score, which may not fully capture the dynamic nature of pain management across different stages of treatment. The number of radiation therapy fractions varied among patients, introducing another layer of complexity and potential variability in pain outcomes. We acknowledge that these factors, along with the exclusion of certain patient groups and variations in healthcare provider training and experience across regions, could have influenced the results. These elements should be carefully considered when interpreting the findings and their application to broader contexts. Finally, our study was based on the analysis of the PMI, the limitations of which have been previously recognized and discussed (6). In particular, although this tool was previously used in analyses of non-neoplastic pain (4–6), it must be recognized that in this context this tool is not entirely suitable. In fact, for patients with pain that is related to active treatment, even in the curative setting, contemporary guidelines (31) stress the importance of using opioids for the management of strong pain. In stark contrast, the guidelines for survivorship pain emphasize the risk of aberrant behaviors and strongly suggest using opioids only as a last resort, and even then, only in patients who have a low risk of abuse behaviors (31).

Therefore, we hypothesize that the percentage of patients with a negative PMI may be influenced, at least in part, by a reluctance to prescribe opioids for non-cancer pain. Unfortunately, our dataset does not enable us to quantify this percentage. Additionally, it is important to consider that this percentage might be higher in countries where, unlike Italy, there is a significant concern regarding an ‘opioid epidemic.’ This factor could affect opioid prescription practices and consequently the management of pain across different healthcare settings.

To further address the limitations of this study, we acknowledge that the sample size, while sufficient for our analyses, may limit the statistical power to detect smaller but clinically relevant effects. Future studies with larger, more diverse cohorts would be valuable in validating and extending our findings. We also recognize the potential for selection bias, as participation was voluntary, and patients with more severe pain or those less engaged in their care may have been underrepresented. Lastly, while our study design provides a snapshot of current practices, a longitudinal approach would better capture the evolution of pain management strategies over time and their outcomes at different treatment stages. These additional considerations further underscore the need for cautious interpretation and the importance of follow-up research to build upon our findings.

Future research should aim to address these limitations, potentially incorporating more objective pain measurement tools and considering multi-national cohorts to enhance the generalizability and applicability of the findings. Furthermore, integrating qualitative methods could provide a more nuanced understanding of the interplay between healthcare provider approaches, patient attitudes, and systemic healthcare factors in the context of pain management in BCa care.

From a practical point of view, considering the availability of national guidelines for the management of cancer pain in Italy (32), largely based on WHO guidelines, the poor adequacy of pain management recorded in this and previous analyses suggests the need to improve the education and continuing training of physicians on this topic, especially in the setting of RT. Furthermore, considering that the worse results demonstrated by our and other studies (5, 15, 16) regarding BCa cancer patients can hardly be justified by a different respect of guidelines by physicians in these subjects, our analysis should stimulate greater attention to this patient population, since we cannot exclude that they have a lower propensity to report to physicians the intensity of their pain.

## Data Availability

The original contributions presented in the study are included in the article/**Supplementary Material**. Further inquiries can be directed to the corresponding author.
