# Supplementary material for: Exploring pain management in breast cancer: key findings from the ARISE study
Source: Front Oncol. 2025 Mar 12;15:1536709. doi: 10.3389/fonc.2025.1536709 (PMC11936917; doi:10.3389/fonc.2025.1536709)
Supplement: Supplementary file 1 [file SupplementaryFile1.pdf]

Supplementary material 1: Case Report Form (CRO)

DATA COLLECTION FORM (CRF)

Date of Completion: ____ /____ /____

Center: __________________________

Availability of Pain Therapy Service at the Center (Yes / No): ______________

Name and Surname of the Data Collector: ____

Patient Numeric Code: ____________ Initials, Name, and Surname of Patient:____

Sex: ☐ M ☐ F Age: ___________

EVALUATION:

☐ First Visit

☐ During Treatment

☐ End of treatment

PURPOSE OF TREATMENT:

☐ Curative

☐ Palliative

ECOG PS:

☐ = 0

☐ = 1

☐ = 2

☐ = 3

☐ = 4

PRIMARY TUMOR: ___________

**01**	**BREAST**
**02**	PROSTATE
**03**	RECTUM
**04**	UTERUS/CERVIX
**05**	LUNG
**06**	HEAD AND NECK
**07**	LYMPHOMA
**08**	STOMACH
**09**	SKIN
**10**	PANCREAS/BILE DUCTS
**11**	SEMINOMA
**12**	SARCOMA
**13**	ANAL CANAL
**14**	BLADDER
**15**	ESOPHAGUS
**16**	MENINGIOMA
**17**	MESOTHELIOMA
**18**	OTHER

STAGE:

☐ Non-metastatic

☐ Metastatic

PAIN:

☐ Neoplastic (clearly caused by cancer)

☐ Non-neoplastic (clearly caused by other conditions/diseases, e.g., mucositis, osteoarthritis, etc.)

☐ Mixed (caused by a combination of the above)

PAIN SEVERITY:

☐ No (VAS/NRS: 0)

☐ Mild (VAS/NRS: 1-4)

☐ Moderate (VAS/NRS: 5-6)

☐ Severe (VAS/NRS: 7-10)

TREATMENT:

☐ No medication

☐ Non-opioids

☐ Weak opioids

☐ Strong opioids

** In cases where multiple categories of drugs are administered simultaneously, the most potent category should be indicated.*
